# A new type of ArsR transcriptional repressor controls transcription of the arsenic resistance operon of *Arsenicibacter rosenii* SM‐1

**DOI:** 10.1002/mlf2.12155

**Published:** 2025-01-19

**Authors:** Yujie Zhang, Wenjun Wu, Ke Huang, Fang‐Jie Zhao

**Affiliations:** ^1^ Jiangsu Key Laboratory for Organic Waste Utilization, Jiangsu Collaborative Innovation Center for Solid Organic Waste Resource Utilization, College of Resources and Environmental Sciences Nanjing Agricultural University Nanjing China

## Abstract

Arsenic is the most common toxic metalloid in the environment. Nearly all organisms have genes for arsenic detoxification. Arsenic detoxification genes are frequently organized in chromosomal or plasmid‐encoded arsenic resistance (*ars*) operons, which are commonly regulated by members of the ArsR transcriptional repressors. To date, three As(III)‐responsive ArsRs with different As(III) binding sites have been identified. Here, we identify a new type of As(III)‐responsive ArsR repressor that has an atypical As(III) binding site and controls transcription of the *ars* operon of *Arsenicibacter rosenii* SM‐1. Our results provide new insights into the classification and evolution relationship of the ArsR transcriptional repressors.

Arsenic (As) is a toxic metalloid present ubiquitously in the environment. The presence of toxic arsenic species such as inorganic arsenate (As(V)) and arsenite (As(III)) imposes a strong selection pressure for microorganisms to evolve resistance mechanisms^1^, ^2^. As(V) is commonly exported after being reduced to As(III)^1^, ^2^. This reductive step is catalyzed by the arsenate reductase (ArsC)^3^. Detoxification of As(III), on the other hand, is achieved via efflux^4^, intracellular chelation (in eukaryotes)^1^, or transformation (e.g., methylation and volatilization)^2^, ^5^. Microbial As(III) methylation is catalyzed by the As(III) *S*‐adenosylmethionine methyltransferase (ArsM), which converts As(III) into the trivalent methylarsenite (MAs(III)) and dimethylarsenite (DMAs(III)) and nontoxic volatile trimethylarsenite (TMAs(III))^6^, ^7^. Although MAs(III) and DMAs(III) are more toxic than As(III), they are unstable and easily oxidized to the relatively nontoxic pentavalent methylarsenate (MAs(V)) and dimethylarsenate (DMAs(V)) in the oxic environment^6^, ^7^. Thus, this biotransformation process has been recognized as a means of As(III) detoxification for aerobes that harbor *arsM* genes^6^, ^7^. However, in the anoxic environment, As(III)‐methylating anaerobes produce highly toxic MAs(III), which is considered to serve as a primordial antibiotic to suppress or kill other MAs(III)‐sensitive microbial communities and confer the producers with a competitive advantage^8^, ^9^. In addition to As(III) biomethylation, MAs(III) can also be the product of microbial MAs(V) reduction. Some microbes such as *Enterobacter* sp. CZ‐1^10^, *Sinorhizobium meliloti* Rm1021^11^, and *Burkholderia* sp. MR1^12^ have been found to possess the ability to reduce MAs(V) under oxic conditions. In response to MAs(III) toxicity, microorganisms adopt ways to evolve a number of detoxification strategies. One common strategy for aerobes is transformation of MAs(III) into less toxic DMAs(V) or trimethyl arsenicals by the ArsM enzyme^13^.

The genes involved in arsenic detoxification are usually organized in *ars* operons^14^. The majority of *ars* operons are regulated by the homodimeric ArsR transcriptional repressors^15^. Three different types of ArsRs with varying As(III) binding sites have been identified^15^, ^16^, ^17^. The first well‐characterized ArsR encoded by *Escherichia coli* plasmid R773 (R773 ArsR) has an N‐terminal As(III) binding site (Type 1 site) composed of three cysteine residues, Cys32, Cys34, and Cys37, in the DNA binding domain of each monomer^15^. Binding of As(III) to R773 ArsR is proposed to induce a conformational change, resulting in dissociation from the promoter region of its target *ars* operon and transcriptional derepression. The second type, represented by *Acidithiobacillus ferrooxidans* AfArsR, has a C‐terminal As(III) binding site (Type 2 site) that is composed of three cysteine residues, Cys95, Cys96, and Cys102, at the ends of antiparallel C‐terminal helices in each subunit that form a dimerization domain^16^. The third ArsR orthologue with a distinct As(III) binding site was identified from *Corynebacterium glutamicum* ^17^. The Type 3 site of CgArsR is formed by three cysteine residues: Cys15 and Cys16 from one monomer and Cys55 from the other^17^. A common property of these As(III)‐responsive ArsRs is the presence of the three‐coordinate As(III) binding site consisting of three cysteine residues located in different positions^15^, ^16^, ^17^, ^18^. However, not all ArsRs contain the three‐coordinate As(III) binding site. For example, Chen et al. reported that SpArsR of *Shewanella putrefaciens* 200 has a structure similar to AfArsR, but lacks the third cysteine residue (Cys102) of the As(III) binding site in AfArsR^18^. They showed that SpArsR responds to MAs(III) but not to As(III); the former requires only two cysteine residues for binding.

Here, we report a novel *ArarsR* gene encoding a new type of As(III)‐responsive ArsR in *Arsenicibacter rosenii* SM‐1, which is an aerobic bacterium in the *Cytophagaceae* family with an extraordinary ability to methylate As(III)^19^. The *ArarsR* gene is located in an *ArarsRMC* operon that also contains an As(III) *S*‐adenosylmethionine methyltransferase gene *ArarsM* and a putative As(V) reductase gene *ArarsC* (Figure 1A). When the *ArarsRMC* operon was expressed in the arsenic‐sensitive *E. coli* strain AW3110, the cells became more tolerant to As(III), As(V), and MAs(III) (Figure S1). The enhanced tolerance to As(III) and MAs(III) is likely through As(III)/MAs(III) methylation catalyzed by ArArsM^19^, whereas the increased tolerance to As(V) probably results from the action of both ArArsC, which reduces As(V) to As(III)^20^, and ArArsM. Promoter analysis revealed that the *ArarsRMC* genes share a common promoter upstream of *ArarsR* (Figure 1A). In addition, reverse transcription PCR analysis showed that the transcription of two intergenic regions in the *ArarsRMC* operon (Figure 1A) was detected in As(III)‐treated cells but not in As(III)‐untreated cells (Figure 1B). These results indicate that all three genes of the *ArarsRMC* operon are co‐transcribed. Furthermore, real‐time quantitative PCR analysis showed that the transcript levels of *ArarsR*, *ArarsM*, and *ArarsC* were induced 158‐, 351‐, and 297‐fold, respectively, by 10 µM As(III) and 23‐, 48‐, 55‐fold, respectively, by 3 µM MAs(III) (Figure 1C), indicating that the expression of the *ArarsRMC* operon is inducible by As(III) and MAs(III). The effect of *ArarsR* on the expression of downstream genes in the *ArarsRMC* operon was also evaluated heterologously in *E. coli*. The relative transcription levels of *ArarsM* and *ArarsC* in *E. coli* AW3110 bearing pBB‐*ArarsRMC* were 4.8‐ and 3.5‐fold, respectively, lower than those in *E. coli* AW3110 bearing pBB‐*ArarsMC* (Figure 1D), suggesting that the presence of *ArarsR* had an inhibitory effect on the transcription of *ArarsM* and *ArarsC* in the same operon. These results all suggest that ArArsR is an As(III)‐ and MAs(III)‐responsive transcriptional repressor, similar to R773 ArsR, AfArsR, and CgArsR found in *E. coli* plasmid R773^15^, *A. ferrooxidans* ^16^, and *C. glutamicum* ^17^.

**Figure 1:**
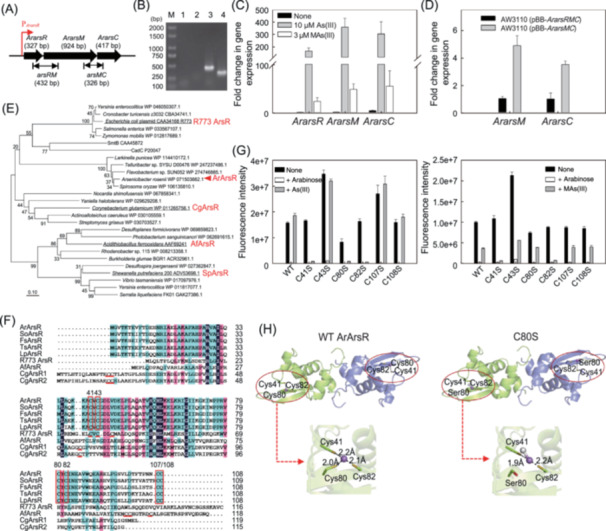
*ArarsR* encodes a new type of As(III)‐responsive ArsR repressor that has an atypical As(III) binding site and regulates the expression of the *ArarsRMC* operon. (A) Organization of the *ArarsRMC* gene cluster. The locations and size of the amplified DNA fragments of *arsRM* and *arsMC* for reverse transcription (RT) PCR analysis are indicated by thin arrows. (B) Analysis of *ArarsRMC* transcription by RT‐PCR. Reactions performed without RT were used as negative controls, as shown in lanes 1 and 2. Lane M, molecular size markers. Lanes 3 and 4 (template from strain SM‐1 induced by 10 µM As(III)) show products amplified using the R_*arsRM*_‐F/R_*arsRM*_‐R and R_*arsMC*_‐F/R_*arsMC*_‐R primer pairs sets, respectively, with the products of RT. (C) RT‐qPCR analysis of the transcriptional levels of genes in the *ArarsRMC* operon in *Arsenicibacter rosenii* SM‐1 cultured in the absence or presence of 10 µM As(III) or 3 µM MAs(III). (D) Comparison of the transcriptional levels of *ArarsM* and *ArarsC* in heterologous expression hosts *E. coli* AW3110 (pBB‐*ArarsRMC*) and *E. coli* AW3110 (pBB‐*ArarsMC*) grown in Km‐containing LB medium. (E) Evolutionary relationships of ArArsR with ArsRs from other bacterial species. The phylogenetic tree constructed using the neighbor‐joining method shows five types of ArsRs with different placements of As(III)‐ or MAs(III)‐binding cysteine residues. ArArsR is indicated by the red triangle. (F) Multiple alignment of ArArsR homologues. Representative ArsR homologues are from *A. rosenii* SM‐1, *Spirosoma oryzae* (SoArsR), *Flavobacterium* sp. SUN052 (FsArsR), *Telluribacter* sp. SYSU D00476 (TsArsR), *Larkinella punicea* (LpArsR), plasmid R773 (R773 ArsR), *Acidithiobacillus ferrooxidans* (AfArsR), and *Corynebacterium glutamicum* (CgArsR1; CgArsR2). The conserved cysteine residues in ArArsR and its four closest homologues are indicated by the red box. The typical As(III) binding sites of the well‐characterized R773 ArsR, AfArsR, CgArsR1, and CgArsR2 are indicated by the red underline. (G) In vivo regulation of *mCherry* expression from the *ArarsR* promoter using wild‐type ArArsR and six Cys mutants (C41S, C43S, C80S, C82S, C107S, and C108S) in the presence or absence of As(III) or MAs(III). Expression of the *mCherry* reporter gene was assayed as described in Supporting Information section. (H) Structure of wild‐type ArArsR with bound As(III) and the C80S mutant with bound MAs(III). Structural models of WT ArArsR and C80S were constructed by the software Modeller using the crystal structure of CmtR (PDB ID: 2JSC) as a template. The two monomers in both structures are colored in cyan and purple, respectively. The cysteine residues are shown in stick representation and are colored in cyan and orange (sulfur atom). The serine residue is shown in stick representation and is colored in cyan and red (hydroxyl group). The purple sphere is the arsenic atom and the gray sphere is the methyl group.

However, phylogenetically, ArArsR forms a cluster that is distinct from other known As(III)‐responsive ArsRs (Figures 1E and S2). It has been suggested that three cysteine residues in As(III)‐responsive ArsRs are required for As(III) binding^16^, ^17^, ^18^. A multiple sequence alignment shows that ArArsR contains six cysteine residues, two in the N‐terminal (Cys41 and Cys43) and four in the C‐terminal domain (Cys80, Cys82, Cys107, and Cys108), which are conserved among the four closest homologues of ArArsR (Figure 1F). ArArsR lacks the typical three‐coordinate Cys95–Cys96–Cys102 As(III) binding site of AfArsR or the Cys15–Cys16–Cys55 binding sites of CgArsR1 and CgArsR2. Cys41 and Cys43 of ArArsR correspond to Cys32 and Cys34 in the As(III) binding site of R773 ArsR, but there is no corresponding Cys in the ArArsR for the third cysteine residue (Cys37) in R773 ArsR, which is required as a ligand for As(III) binding. These comparisons suggest that ArArsR has an atypical three‐coordinate As(III) binding site. To further examine the arsenic binding affinity of ArArsR, a two‐plasmid *mCherry* reporter biosensor was constructed by introducing pBAD‐*ArarsR* and pBB‐P_*ArArsR*_‐*mCherry* into *E. coli* strain AW3110 (Figure S3A). The expression of *ArarsR* and *mCherry* in these two plasmids are under the control of the arabinose promoter and the *ArarsR* promoter, respectively. The expression of *mCherry* was constitutive when *ArarsR* was repressed in the absence of arabinose in the growth medium (Figure S3B). When ArArsR was expressed by the addition of 0.2% arabinose, *mCherry* was repressed and mCherry fluorescence was not produced by the biosensor cells. The addition of As(III) or MAs(III) induced an increase in *mCherry* expression and the production of fluorescence by derepressing ArArsR (Figure S3C). In contrast, the addition of As(V), MAs(V), or DMAs(V) did not induce *mCherry* expression (Figure S3C). These results indicate that ArArsR is an As(III)‐ and MAs(III)‐sensing repressor, which does not respond to pentavalent As(V), MAs(V), and DMAs(V). Next, we examined the cysteine residues in ArArsR for As(III) and MAs(III) binding by mutating the *ArarsR* gene in plasmid pBAD‐*ArarsR* that was used to construct the two‐plasmid *mCherry* reporter biosensor. Mutation of Cys43, Cys107, or Cys108 in ArArsR individually to serine did not decrease the responsiveness of the biosensor to the addition of As(III) or MAs(III) compared with wild‐type ArArsR, suggesting that these three cysteine residues are not required for As(III) or MAs(III) binding (Figure 1G). The C41S and C82S mutants lost the responsiveness to the addition of As(III) or MAs(III). The C80S mutant lost the ability to respond to As(III), but still retained the ability to respond to MAs(III) (Figure 1G). These results indicate that Cys41, Cys80, and Cys82 are required for As(III) binding, while Cys41 and Cys82 are also required for MAs(III) binding. Homology modeling of the ArArsR structure suggests that the As(III) binding site in each monomer is formed between Cys41 in the N‐terminal loop region and Cys80 and Cys82 in the C‐terminal β‐sheet region (Figure 1H). When Cys80 is mutated to serine, the mutant (C80S) is unable to bind As(III) but is still able to form a two‐coordinate MAs(III) binding site (Figure 1H), because MAs(III) requires only two ligands for binding. It has been reported that the As(III) binding sites of R773 ArsR, AfArsR, and CgArsR have ligands that are located at the beginning or the end of α helices, unraveling these helices results in dissociation of these three ArsRs from their own promoter DNA^17^. This may not be the full mechanism of derepression in ArArsR because of the different locations of the three cysteine residues for As(III) binding. Only one of the three cysteine residues, Cys41, in the ArArsR As(III) binding site corresponds to an essential cysteine residue (Cys32) in the R773 ArsR. On the other hand, although ArArsR Cys43 corresponds to Cys34 in the As(III) binding site of R773 ArsR, it is not essential for As(III) binding. Furthermore, none of the cysteine residues in ArArsR aligns with those in the other two known As(III)‐responsive ArsRs from *A. ferrooxidans* and *C. glutamicum*. Thus, ArArsR should be classified as representing a new type of ArsR repressor that regulates transcription of the *ars* operon. It is highly likely that the As(III) binding site in ArArsR has evolved independently of the As(III) binding sites in AfArsR or CgArsR. Since the As(III) binding sites of ArArsR and R773 ArsR are partially related to each other, they may have evolved from a common ancestor.

We identified an ArArsR‐protected sequence in the promoter region of its own gene as the binding site for ArArsR. The DNase I footprinting and EMSA assays (Figures S4 and S5) revealed that the ArArsR repressor could interact with the DNA located at −46 to −19 nucleotides (5′‐TTTTATCGTAATATTGCGATCACAATC‐3′) upstream of the start codon of the *ArarsR* gene, suggesting that the ArArsR binding site overlaps with the −35 element of the *ArarsR* promoter. It is expected that ArArsR binding would block or interfere with the initiation of transcription of the *ArarsRMC* operon, similar to the action of other four ArsRs identified to date^15^, ^16^, ^17^, ^18^. We propose an As(III)‐ and MAs(III)‐sensing and adaptive regulation model for the *ArarsRMC* operon in *A. rosenii* SM‐1. In the absence of As(III) or MAs(III), ArArsR binds to the promoter of its own gene and prevents RNA polymerase from initiating transcription of the *ArarsRMC* operon. In the presence of As(III) or MAs(III), ArArsR binds the arsenicals and produces a conformational change in the N‐terminal loop and C‐terminal β‐sheet regions, resulting in the release of the ArArsR repressor from the promoter to initiate transcription of the *ArarsRMC* operon and enhanced detoxification of As(III) and MAs(III).

In summary, we have identified a new type of As(III)‐responsive ArsR repressor that regulates the transcription of the *ars* operon in *A. rosenii* SM‐1. This new type of repressor is common among the families *Cytophagaceae*, *Spirosomaceae*, and *Flavobacteriaceae*. The different types of ArsRs likely arise through convergent and parallel evolution of As(III) binding sites on the common backbone of the winged helix repressors. Our findings provide new insight into the classification and evolutionary relationship of the ArsR transcriptional repressors, which are the master regulators for the resistance to highly toxic trivalent arsenicals in the environment.

## AUTHOR CONTRIBUTIONS

**Yujie Zhang**: Investigation (lead). **Wenjun Wu**: Investigation (supporting). **Ke Huang**: Conceptualization (lead); formal analysis (lead); funding acquisition (lead); investigation (supporting); supervision (lead); and writing—original draft (lead). **Fang‐Jie Zhao**: Funding acquisition (supporting); supervision (supporting); and writing—review and editing (lead).

## ETHICS STATEMENT

No animal or human research was involved in this study.

## CONFLICT OF INTERESTS

The authors declare no conflict of interests.

## Supporting information

Supporting information.

## Data Availability

The draft genome and sequences of *ArarsR*, *ArarsM*, and *ArarsC* of *Arsenicibacter rosenii* SM‐1 have been submitted to the GenBank database under the accession numbers MORL00000000, OR208258, KU641426.1, and OR208259.
