# Supplementary material for: A new type of ArsR transcriptional repressor controls transcription of the arsenic resistance operon of Arsenicibacter rosenii SM‐1
Source: mLife. 2025 Jan 19;4(1):96–100. doi: 10.1002/mlf2.12155 (PMC11868830; doi:10.1002/mlf2.12155)
Supplement: Supplementary file 1 — Supporting information. [file MLF2-4-96-s001.docx]

**A** **new type of ArsR** **transcriptional repressor controls transcription of the arsenic resistance operon of *Arsenicibacter rosenii* SM-1**

Yu-Jie Zhang^1^, Wen-Jun Wu^1^, Ke Huang^1,*^, and Fang-Jie Zhao^1^

^1^Jiangsu Key Laboratory for Organic Waste Utilization, Jiangsu Collaborative Innovation Center for Solid Organic Waste Resource Utilization, College of Resources and Environmental Sciences, Nanjing Agricultural University, Nanjing, China.

*Corresponding author: Ke Huang, Email: [kehuang518@njau.edu.cn](mailto:Fangjie.Zhao@njau.edu.cn)

**MATERIALS AND METHODS**

**Chemicals and media**

Unless otherwise indicated, all reagents were purchased from commercial sources. As(III), As(V) and DMAs(V) were obtained from Sigma-Aldrich (Mainland, China). MAs(V) was obtained from Sunlida (Nanjing, China). MAs(III) was prepared according to the method previously reported^1^. R2A medium used in this study consists of the following components: 0.5 g L^-1^ yeast extract, 0.5 g L^-1^ tryptone, 0.5 g L^-1^ casamino acid, 0.5 g L^-1^ D-glucose, 0.5 g L^-1^ starch, 0.3 g L^-1^ K_2_HPO_4_, 0.05 g L^-1^ MgSO_4_·7H_2_O, 0.3 g L^-1^ sodium pyruvate. LB medium consists of 10 g L^-1^ tryptone, 5 g L^-1^ yeast extract, and 10 g L^-1^ NaCl. 20×ST10^-1^ medium consists of 10 g L^-1^ tryptone, and 1 g L^-1^ yeast extract.

**Strains, plasmids, primers, and culture conditions**

The bacterial strains and plasmids used in this study are presented in Table S1. The primers used in this study are presented in Table S2. *Arsenicibacter rosenii* SM-1 was grown at 37^o^C in R2A medium or R2A agar. Unless otherwise specified, *E. coli* strains were grown at 37^o^C in LB medium or LB agar. Where required, antibiotics were used at the following concentrations: ampicillin (Amp), 100 µg mL^-1^; and kanamycin (Km), 50 µg mL^-1^.

**DNA manipulation**

DNA manipulations used in this study were performed according to standard procedures^2^. Genomic DNA was extracted using the CTAB method^3^. Plasmid extraction and DNA purification were performed using a Plasmid Mini Kit I (Omega, USA) and a Gel Extraction Kit (Omega, USA). DNA polymerases, homologous recombinase, restriction enzymes and T4 DNA ligase were purchased from Vazyme (Nanjing, China), Genesand (Nanjing, China), New England BioLabs (Beijing) LTD., and Takara (Japan), respectively. Transfer of plasmid vectors into *E. coli* strains was accomplished by transformation^2^. DNA sequencing was performed by Tsingke (Nanjing, China).

**Phylogenetic analysis**

Multiple sequence alignment was performed with the sequences of ArArsR and other eight representative ArsR homologs using DNAMAN software. Acquisition of sequences was carried out by searching a list of reference organisms or from the NCBI (National Center for Biotechnology Information) protein database using a Protein BLAST search. Phylogenetic analysis was performed to infer the evolutionary relationship among the ArsR proteins of various organisms. The phylogenetic trees were constructed using the neighbor-joining and the minimum-evolution methods using MEGA 7.0 software. The statistical significance of the branch pattern was estimated by a bootstrap analysis with 1000 replicates.

**Plasmid construction and mutagenesis**

To express *ArarsRMC* operon from *Arsenicibacter rosenii* SM-1 in *E. coli*, plasmid pBB-*ArarsRMC* was constructed in which the gene cluster *ArarsRMC* is under the control of the *ArarsR* promoter. The *ArarsRMC* operon was cloned from the genomic DNA of strain SM-1 using the primers *ArarsRMC*-F and *ArarsRMC*-R and ligated with the framework fragment amplified from the plasmid pBBR1MCS using the primers pBBR1MCS-F and pBBR1MCS-R to generate the plasmid pBB-*ArarsRMC*. The upstream and downstream fragments of *ArarsR* within the *ArarsRMC* operon were [respectively](https://fanyi.sogou.com/?keyword=%20respectively&fr=websearch_submit&from=en&to=zh-CHS) amplified using the primer pairs *ArarsRMC*-F/*ArarsMC*-F-R and *ArarsMC*-R-F/*ArarsRMC*-R. The two PCR products containing the *ArarsR* promoter and the expression regions of the *ArarsM* and *ArarsC* genes were spliced by overlap extension to generate the fragment P*_ArarsR_*+*ArarsMC*. The fusion product was then ligated into the plasmid pBBR1MCS to generate plasmid pBB-*ArarsMC*.

To express *ArarsR* in *E. coli*, the encoding region of *ArarsR* excluding the stop codon was amplified using the primers *WTArarsR*-F and *WTArarsR*-R. The PCR product was gel purified and digested with *Nde*I and *Xho*I and then cloned into vector pET29a(+) that had been digested using *Nde*I and *Xho*I, generating plasmid pET29a-*ArarsR_WT_*.

To construct plasmid pClone007-P*_ArarsR_* used as the template for preparation of fluorescent 6-carboxyfluorescein (FAM)-labelled probes in DNase I footprinting assay, a 248-bp promoter region of *ArarsR* was amplified using the primers P*_ArarsR_*-F and P*_ArarsR_*-R and then cloned into pClone007 Blunt vector.

To examine the binding affinity of ArArsR to different arsenicals, a two-plasmid *mCherry* reporter biosensor *E. coli* AW3110 (pBAD-*ArarsR_WT_*/pBB-P*_ArArsR_*-*mCherry*) was constructed. In plasmid pBAD-*ArarsR_WT_*, *ArarsR* gene was under the control of the arabinose promoter. The *ArarsR* gene was cloned using the primers *ArarsR_WT_*-F and *ArarsR_WT_*-R. The PCR product was digested with *Nco*I and *Sal*I and then cloned into *Nde*I/*Xho*I-digested vector pBAD/myc-HisA, generating plasmid pBAD-*ArarsR_WT_*. In plasmid pBB-P*_ArArsR_*-*mCherry*, *mCherry* gene is under the control of the *ArarsR* promoter. The fragments of the *ArarsR* promoter and *mCherry* gene were [respectively](https://fanyi.sogou.com/?keyword=%20respectively&fr=websearch_submit&from=en&to=zh-CHS) amplified from *A. rosenii* genomic DNA and plasmid pUC57-Tac-*mCherry* using the primer pairs P*_ArarsR_*-MC-F/P*_ArarsR_*-MC-R and *mCherry*-F/*mCherry*-R. The two PCR products were spliced by overlap extension to generate the fragment P*_ArarsR_*+*mCherry*. The fusion fragment was ligated into the plasmid pBBR1MCS to generate plasmid pBB-P*_ArArsR_*-*mCherry*.

Mutants of the wild-type ArArsR protein were generated by site-directed mutagenesis. Four cysteine-to-serine mutants (C41S, C43S, C80S and C82S) were generated using overlap extension PCR according to the method of Ho et al.^4^. The primer pairs used to introduce these mutations includes (i) *ArarsR_WT_*-F/*ArarsR_C41S_*-F-R, and *ArarsR_C41S_*-R-F/*ArarsR_WT_*-R, (ii) *ArarsR_WT_*-F/*ArarsR_C43S_*-F-R, and *ArarsR_C43S_*-R-F/*ArarsR_WT_*-R, (iii) *ArarsR_WT_*-F/*ArarsR_C80S_*-F-R, and *ArarsR_C80S_*-R-F/*ArarsR_WT_*-R, (iv) *ArarsR_WT_*-F*/ArarsR_C82S_*-F-R, and *ArarsR_C82S_*-R-F/*ArarsR_WT_*-R. The C107S and C108S mutants were generated using conventional PCR with the primer pairs *ArarsR_WT_*-F/*ArarsR_C107S_*-R and *ArarsR_WT_*-F/*ArarsR_C108S_*-R, [respectively](https://fanyi.sogou.com/?keyword=%20respectively&fr=websearch_submit&from=en&to=zh-CHS). Each mutation was confirmed by DNA sequencing. The fragments of six cysteine-to-serine mutants were digested with *Nco*I and *Sal*I and cloned separately into pBAD/myc-HisA to generate plasmids pBAD-*ArarsR_C41S_*, pBAD-*ArarsR_C43S_*, pBAD-*ArarsR_C80S_*, pBAD-*ArarsR_C82S_*, pBAD-*ArarsR_C107S_* and pBAD-*ArarsR_C108S_*.

**Arsenic resistance assays**

Single colonies of *E. coli* AW3110 bearing plasmid pBB-*ArarsRMC* or vector plasmid pBBR1MCS2 were inoculated into 4 ml of LB medium supplemented with Km and incubated at 37 ^o^C overnight. Overnight cultures were diluted to an OD_600_ (Optical density at 600 nm) of 0.01 in Km-supplemented LB medium or 20×ST10^-1^ medium with the indicated concentrations of As(III), As(V) or MAs(III). Growth was performed at 37 ^o^C in a shaker with 200 rpm and monitored as OD_600_ at the indicated time point.

**Reverse transcription PCR and real-time quantitative PCR**

To determine whether the genes *ArarsR*, *ArarsM* and *ArarsC* were co-transcribed and induced by As(III), cells of *Arsenicibacter rosenii* SM-1 were cultured at 37^o^C in R2A medium with or without 10 µM As(III) or 3 µM MAs(III). Total RNA was extracted from cells in mid-exponential phase (OD_600_≈0.8) using a Bacterial RNA kit (Omega). DNase digestion and reverse transcription (RT) were performed using a HiScript II Q Select RT SuperMix for qPCR kit (Vazyme), and the resulting cDNA was used to amplify the intergenic regions of the *ArarsR*-*ArarsM*-*ArarsC* genes with the following primer pairs: R*_arsRM_*-F/R*_arsRM_*-R and R*_arsMC_*-F/R*_arsMC_*-R. RT-PCR reactions performed using the total RNA without RT as the template were set as negative controls. Real-time quantitative PCR (RT-qPCR) was conducted with SYBR green Master Mix (Vazyme) to quantify the transcripts of the *ArarsR*, *ArarsM* and *ArarsC* genes. Reactions were performed in a CFX96 thermocycler system (Bio-Rad) with the primer pairs D*_ArarsR_*-F/D*_ArarsR_*-R, D*_ArarsM_*-F/D*_ArarsM_*-R, and D*_ArarsC_*-F/D*_ArarsC_*-R. A 255-bp fragment of the *ArgyrB* gene of strain SM-1 amplified using the primer pair D*_ArgyrB_*-F/D*_ArgyrB_*-R was used as the reference. Post-PCR melting curve analysis was used to evaluate amplification specificity. Relative fold changes in target gene expression were calculated by using the 2^-ΔΔCT^ method.

To examine whether ArArsR is involved in the regulation of the *ArarsRMC* operon, the entire operon and the *ArarsR*-excluding derivative (*ArarsMC*) were cloned and expressed heterologously in *E. coli* strain AW3110. The transcriptional levels of *ArarsM* and *ArarsC* in *E. coli* AW3110 bearing plasmid pBB-*ArarsRMC* or pBB-*ArarsMC* were determined using RT-qPCR. Both *E. coli* strains were grown to mid-exponential phase (OD_600_≈0.6) at 37^o^C in Km-containing LB medium. Total RNA extracted from each of the cultures was used as the template to generate the target cDNA. The primers for RT-qPCR were D*_ArarsM_*-F and D*_ArarsM_*-R for the *ArarsM* gene, D*_ArarsC_*-F and D*_ArarsC_*-R for the *ArarsC* gene, D*_EcgyrB_*-F and D*_EcgyrB_*-R for the reference gene (*E. coli* *gyrB*). The relative gene expression levels were calculated using the method described above.

**DNase I footprinting assay**

For preparation of fluorescent FAM-labeled probe used in DNase I footprinting experiments, the promoter region of *ArarsR* was PCR amplified from the plasmid pClone007-P*_ArarsR_* using the primers M13F (FAM) and M13R (FAM). The purified FAM-labeled probe was quantified by using a Nanodrop-2000c spectrophotometer (Thermo Scientific). DNase I footprinting assays were performed using the method as described previously^5^.

**Overexpression and purification of ArArsR**

Wild-type ArArsR was over-expressed in *E. coli* strain BL21 (DE3). To induce expression of His-tagged ArArsR, cells of *E. coli* BL21 bearing plasmid pET29a-*ArarsR_WT_* were grown with shaking at 200 rpm at 37^o^C in 100 mL of LB medium supplemented with Km. When the culture reached an OD_600_ of 0.6, 0.3 mM Isopropyl β-D-1-Thiogalactoside (IPTG) was added to induce expression of ArArsR. After induction at 37^o^C for another 4 h, the culture was harvested by centrifugation at 13,500 *g* for 10 min, washed twice at 4 ^o^C with 20 mL of buffer A (50 mM MOPS, 20% (v/v) glycerol, 500 mM NaCl, 10 mM 2-mercaptoethanol, 20 mM imidazole, pH=7.5), and then suspended in 20 mL of buffer A. Suspended cells were lysed by an ultrasonic cell disruption (Xianou, China). Cell debris was removed by centrifugation at 13,500 *g* at 4^o^C for 45 min. The supernatant was loaded onto a 1 ml Ni-NTA column (Sangon, China) preequilibrated with 20 mL of buffer A. Protein elution was performed with 4 mL of buffer B (50 mM MOPS, 20% (v/v) glycerol, 500 mM NaCl, 10 mM 2-mercaptoethanol, 200 mM imidazole, pH=7.5). Purified ArArsR was identified by sodium-dodecyl-sulfate polyacrylamide gel electrophoresis (SDS-PAGE). Protein concentration was measured on a Nanodrop-2000c spectrophotometer (Thermo Scientific).

**Electrophoretic mobility shift assays**

A 27-bp *ArarsR* promoter DNA probe obtained from DNase I footprinting assays was used for electrophoretic mobility shift assay (EMSA). The biotin-labelled promoter probe was synthesized through annealing primer pair EMSA-F/EMSA-R labelled on each of the 5’ ends with biotin. EMSA was carried out with a chemiluminescent EMSA Kit (Beyotime, China) using the same method described by Zhang et al.^5^.

**Assay of arsenic binding in vivo**

Single colonies of *E. coli* AW3110 (pBB-P*_ArArsR_*-*mCherry*) bearing plasmid pBAD-*ArarsR_WT_*, pBAD-*ArarsR_C41S_*, pBAD-*ArarsR_C43S_*, pBAD-*ArarsR_C80S_*, pBAD-*ArarsR_C82S_*, pBAD-*ArarsR_C107S_* or pBAD-*ArarsR_C108S_* were separately inoculated into 25 mL of LB medium supplemented with Amp and Km and incubated at 37^o^C overnight. Overnight cultures were diluted 50-fold into 25 mL of LB medium containing Amp and Km and induced at 30^o^C for 24 h with 0.2% arabinose to sufficiently express wild-type ArArsR or other cysteine-to-serine mutants. For constitutive expression of mCherry while repressing ArArsR expression, 0.2% glucose was used instead of arabinose. For arsenic induction assays, 0.2% arabinose and different arsenicals were simultaneously added for 24 h. Expression of mCherry was monitored by measuring the fluorescence of *E. coli* cells using a microplate spectrophotometer (Molecular Devices, America) with an excitation wavelength of 587 nm and an emission wavelength of 630 nm.

**Construction of homology models**

Homology models were constructed using the protein structure homology modeling software MODELLER^6^. Model quality was estimated on the basis of the value of the MODELLER objective function. The models of wild-type ArArsR and the C80S mutant were built using the crystal structure of CmtR from *Mycobacterium tuberculosis* (PDB ID: 2JSC) as the template. *In silico* As(III) was docked to the indicated cysteine residues of wild-type ArArsR to build the As(III)-bound ArArsR model using the AutoDock software^7^. The model of MAs(III)-bound C80S mutant was constructed using the same method. The crystal structures of AfArsR and CgArsR are available in the protein data bank under the accession number 6J05 and 6J0E respectively^8^. R773 ArsR and SpArsR homology models were built using the crystal structure of RevM (PDBs ID: 6JM2) and AfArsR (PDBs ID: 6J05) respectively, as the templates. PyMOL v2.0 was used to visualize the structural models^9, 10^ (<http://www.pymol.org/citing>).

**Table S1** Strains and plasmids used in this study

| **Strain or plasmid** | **Characteristic(s)*^a^*** | **Reference or source** |
| --- | --- | --- |
| **Strains** |  |  |
| *Arsenicibacter rosenii* SM-1 | Wild type; an efficient As(III)-methylating bacterium | ^11, 12^ |
| *E. coli* Trans5α | F^-^ φ80*lacZ*ΔM15 Δ(*lacZYA*-*argF*) U169 *endA1* *recA1* *hsdR17*(rk^-^, mk^+^) | TransGen |
| *E. coli* BL21(DE3) | F^-^ *ompT* hsdS (r_B_^-^ m_B_^-^) *gal* *dcm lacY1* (DE3) | Takara |
| *E. coli* AW3110 | Arsenic-hypersensitive strain of *E. coli* lacking the chromosomal *arsRBC* operon | ^13^ |
|  |  |  |
| **Plasmids** |  |  |
| pET29a(+) | Km^r^; expression vector | Takara |
| pET29a-*ArarsR_WT_* | Km^r^; pET29a(+) harboring *ArarsR_WT_* | This study |
| pUC57-Tac-*mCherry* | Amp^r^; pUC57 harboring *mCherry* | Lab stock |
| pBBR1MCS-2 | Km^r^; broad-host-range cloning vector | ^14^ |
| pBB-*ArarsRMC* | Km^r^; pBBR1MCS-2 harboring the *ArarsRMC* operon | This study |
| pBB-*ArarsMC* | Km^r^; pBBR1MCS-2 harboring the *ArarsRMC* operon excluding the *ArarsR* gene | This study |
| pClone007 Blunt vector | Amp^r^; *E. coli* cloning vector | Tsingke |
| pClone007-P*_ArarsR_* | Amp^r^; pClone007 Blunt vector containing the promoter region of *ArarsR* | This study |
| pBB-P*_ArArsR_*-*mCherry* | Km^r^; pBBR1MCS-2 containing the fragment P*_ArarsR_* + *mCherry* | This study |
| pBAD/myc-HisA | Amp^r^; expression vector | MiaoLing plasmid platform |
| pBAD-*ArarsR_WT_* | Amp^r^; pBAD/myc-HisA harboring *ArarsR_WT_* | This study |
| pBAD-*ArarsR_C41S_* | Amp^r^; pBAD/myc-HisA harboring *ArarsR_C41S_* | This study |
| pBAD-*ArarsR_C43S_* | Amp^r^; pBAD/myc-HisA harboring *ArarsR_C43S_* | This study |
| pBAD-*ArarsR_C80S_* | Amp^r^; pBAD/myc-HisA harboring *ArarsR_C80S_* | This study |
| pBAD-*ArarsR_C82S_* | Amp^r^; pBAD/myc-HisA harboring *ArarsR_C82S_* | This study |
| pBAD-*ArarsR_C107S_* | Amp^r^; pBAD/myc-HisA harboring *ArarsR_C107S_* | This study |
| pBAD-*ArarsR_C108S_* | Amp^r^; pBAD/myc-HisA harboring *ArarsR_C108S_* | This study |
| *^a^*Km^r^, kanamycin resistance, Amp^r^, ampicillin resistance. | | |

**Table S2** Primers used in this study

| **Primer** | **Sequence*^a^*** |
| --- | --- |
| Reverse transcription PCR | |
| R*_arsRM_*-F | GCAGGCAACTGTCTCGCAGCATC |
| R*_arsRM_*-R | TTGGTGGGCAAGCCGCATC |
| R*_arsMC_*-F | ATGACATCCTTCAGCATTATCTTTCG |
| R*_arsMC_*-R | CAACGCCCGCACTGTATACCTC |
|  |  |
| Real-time quantitative PCR | |
| D*_ArarsR_*-F | CCGAAGTTTTTACCGACGAGCAT |
| D*_ArarsR_*-R | ATAGCACACCCGTGGGGGATTTA |
| D*_ArarsM_*-F | TCTGGTGCCGAATAAGAAAAATGTT |
| D*_ArarsM_*-R | CCGAAAGATAATGCTGAAGGATGTC |
| D*_ArarsC_*-F | GTCAAACCATGTAGATGAATATCAGCAC |
| D*_ArarsC_*-R | TTAGACAATCGTTTCGTTTACAAAGTTC |
| D*_ArgyrB_*-F | TTCCAGGCTATTCTGCCGCTT |
| D*_ArgyrB_*-R | TAACCGCAGGTGACGGAAGAAC |
| D*_EcgyrB_*-F | CAGGAAAACATCTACTGCTTTACCAACAAC |
| D*_EcgyrB_*-R | GCTGACTTTGGCTTTTTTGCTGTAG |
|  |  |
| Construction of the plasmids pBB-*ArarsRMC* and pBB-*ArarsMC* | |
| *ArarsRMC*-F | AAGCTTGATATCGAATTCCTAAAGAATGAATAATGAATAATGGGTAATGAAT |
| *ArarsRMC*-R | CTAGAACTAGTGGATCCCCCACCGGGCTGGTCGTGGCAT |
| *ArarsMC*-F-R | GTTTATTGTAATAATACGATAGATATGGGTAAAAAAAGCTGAATGATTGAGTTAATGATTGTGAT |
| *ArarsMC*-R-F | ATCACAATCATTAACTCAATCATTCAGCTTTTTTTACCCATATCTATCGTATTATTACAATAAAC |
| pBBR1MCS-F | AGGAATTCGATATCAAGCTTATCGAT |
| pBBR1MCS-R | GGGGGATCCACTAGTTCTAGAGCG |
|  |  |
| Cloning and expression of *ArarsR_WT_* | |
| *WTArarsR*-F | GGGAATTC**CATATG**GGCGTCACCAAAACCGAAG |
| *WTArarsR*-R | CCG**CTCGAG**ACAGCAGTTGTTAGGGGTAAATGTGTC |
|  |  |
| DNase I footprinting assay | |
| M13F | TGTAAAACGACGGCCAGT |
| M13R | CAGGAAACAGCTATGACC |
| P*_ArarsR_*-F | TAATCCCAGTCATCGGAAGGAGCAC |
| P*_ArarsR_*-R | CAAAGGCCTTGGCCAGCTCC |
|  |  |
| Electrophoretic mobility shift assay | |
| EMSA-F | TTTTATCGTAATATTGCGATCACAATC |
| EMSA-R | GATTGTGATCGCAATATTACGATAAAA |
|  |  |
| Construction of the plasmids pBBR1MCS-P*_ArArsR_*-mCherry and pBAD-*ArarsR_WT_* | |
| P*_ArarsR_*-MC-F | AAGCTTGATATCGAATTCCTAAAGAATGAATAATGAATAATGGGTAATGAAT |
| P*_ArarsR_*-MC-R | CCTCGCCCTTGCTCACCATGCTGAATGATTGAGTTAATGATTGTGAT |
| *mCherry*-F | ATCACAATCATTAACTCAATCATTCAGCATGGTGAGCAAGGGCGAGG |
| *mCherry*-R | CTAGAACTAGTGGATCCCCCTTACTTGTACAGCTCGTCCATGCC |
| *ArarsR_WT_*-F | CATG**CCATGG**GCGTCACCAAAACCGAAG |
| *ArarsR_WT_*-R | ACGC**GTCGAC**ACAGCAGTTGTTAGGGGTAAATGTGTC |
|  |  |
| Site-directed mutagenesis | |
| *ArarsR_C41S_*-F-R | CGACCAGATCACCGCAGACGGACGCTTTTTTCTGTGCCAGCAACTG |
| *ArarsR_C41S_*-R-F | CAGTTGCTGGCACAGAAAAAAGCGTCCGTCTGCGGTGATCTGGTCG |
| *ArarsR_C43S_*-F-R | CAACTCATCGACCAGATCACCGGAGACGCACGCTTTTTTCTGTGCC |
| *ArarsR_C43S_*-R-F | GGCACAGAAAAAAGCGTGCGTCTCCGGTGATCTGGTCGATGAGTTG |
| *ArarsR_C80S_*-F-R | CCAGACAGCTTCATTGATGCAATAGGACACCCGTGGGGGATTTATTTCG |
| *ArarsR_C80S_*-R-F | CGAAATAAATCCCCCACGGGTGTCCTATTGCATCAATGAAGCTGTCTGG |
| *ArarsR_C82S_*-F-R | CCTGCCAGACAGCTTCATTGATGGAATAGCACACCCGTGGGGGATTTAT |
| *ArarsR_C82S_*-R-F | ATAAATCCCCCACGGGTGTGCTATTCCATCAATGAAGCTGTCTGGCAGG |
| *ArarsR_C107S_*-R | ACGC**GTCGAC**ACAGGAGTTGTTAGGGGTAAATGTGTCAAGCAC |
| *ArarsR_C108S_*-R | ACGC**GTCGAC**AGAGCAGTTGTTAGGGGTAAATGTGTCAAG |
| *^a^*Restriction sites are indicated in boldface. | |


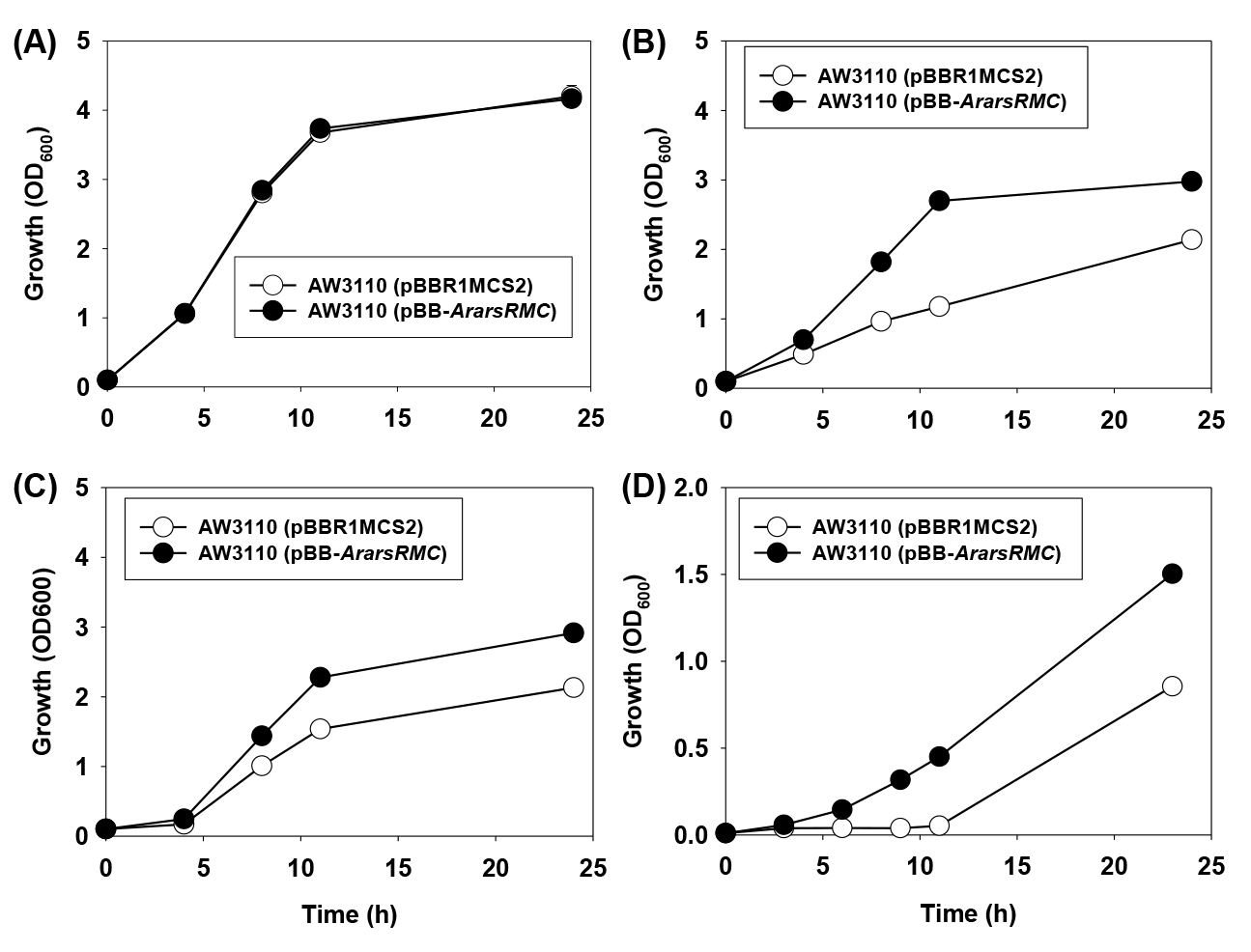
**Fig. S1** Expression of the *ArarsRMC* operon confers resistance to As(III), As(V) and MAs(III) in *E. coli.* Overnight cultures of *E. coli* AW3110 bearing vector plasmid pBBR1MCS2 or plasmid pBB-*ArarsRMC* were diluted to an OD_600_ of 0.01 in LB medium with no As treatment (A), 70 µM As(III) (B) or 1 mM As(V) (C), or in 20×ST10^-1^ medium with 1 µM MAs(III) (D). Growth was carried out at 37 ^o^C with shaking at 200 rpm and measured at the indicated time points. Data are means ± SD (n = 3).


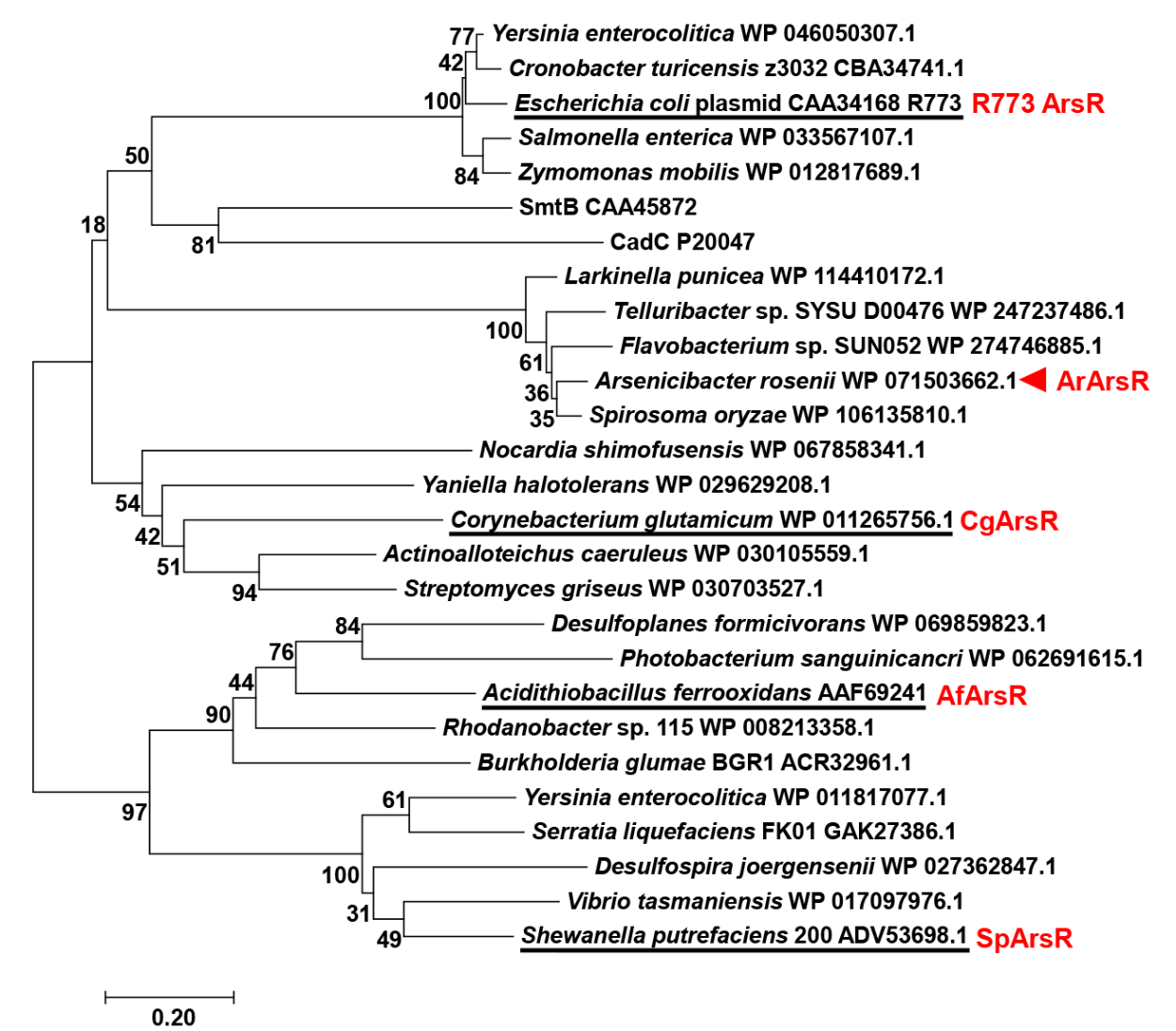
**Fig. S2** Evolutionary relationships of ArArsR with ArsRs from other bacterial species. The phylogenetic tree constructed using the minimum-evolution method shows five types of ArsRs with different placement of As(III)- or MAs(III)-binding cysteine residues. ArArsR is indicated by red triangle.

**Fig. S3** Responses of a bacterial biosensor with ArArsR to As(III) and MAs(III). (A) Diagrammatic representation of the construction of the bacterial biosensor AW3110 (pBAD-*ArarsR*/pBB-P*_ArarsR_*-*mCherry*). In pBAD-*ArarsR*, *ArarsR* is under the control of the *ara* promoter. In pBB-P*_ArarsR_*-*mCherry*, the *mCherry* reporter is under the control of the *ArarsR* promoter. (B) Conditions for constitutive, repressed or derepressed *mCherry* expression. In cells of *E. coli* AW3110 with both plasmids, *ArarsR* is not expressed in the absence of arabinose, and *mCherry* expression is constitutive, producing cellular fluorescence. In the presence of arabinose, *ArarsR* is expressed, and *mCherry* is repressed, so the cells are not fluorescent. In the presence of both arabinose and arsenical inducer, *mCherry* expression is derepressed, and the cells are fluorescent. (C) The bacterial biosensor with *ArarsR* responds to As(III) and MAs(III). Expression of the *mCherry* reporter gene was assayed as described under *EXPERIMENTAL PROCEDURES*. Cells of *E. coli* biosensor were grown without arabinose, 0.2% arabinose, or 0.2% arabinose and arsenicals at the indicated concentrations. Fluorescence intensities were quantified using a microplate spectrophotometer. Data are means ± SD (n = 4).


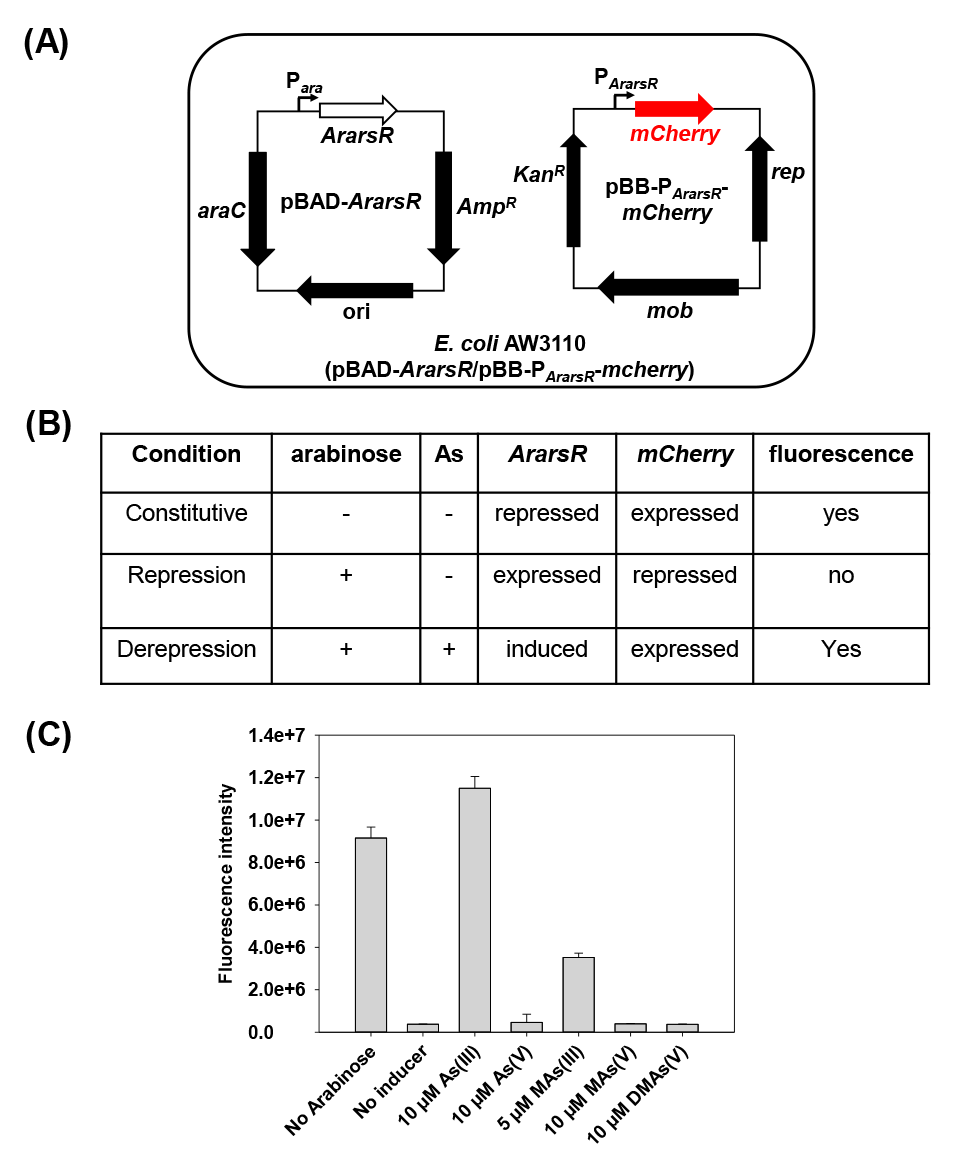


**Fig. S4** Identification of the ArArsR-protected region in the *ArarsR* own promoter region using a DNase I footprinting assay. The probe covering the promoter region of *ArarsR* was labeled with 6-carboxyfluorescein (FAM) dye and incubated with (blue line) or without (red line) 2 µg ArArsR. The region protected by ArArsR from DNase I cleavage is indicated by a black dotted box. The sequence of the ArArsR protected region is shown at the bottom.


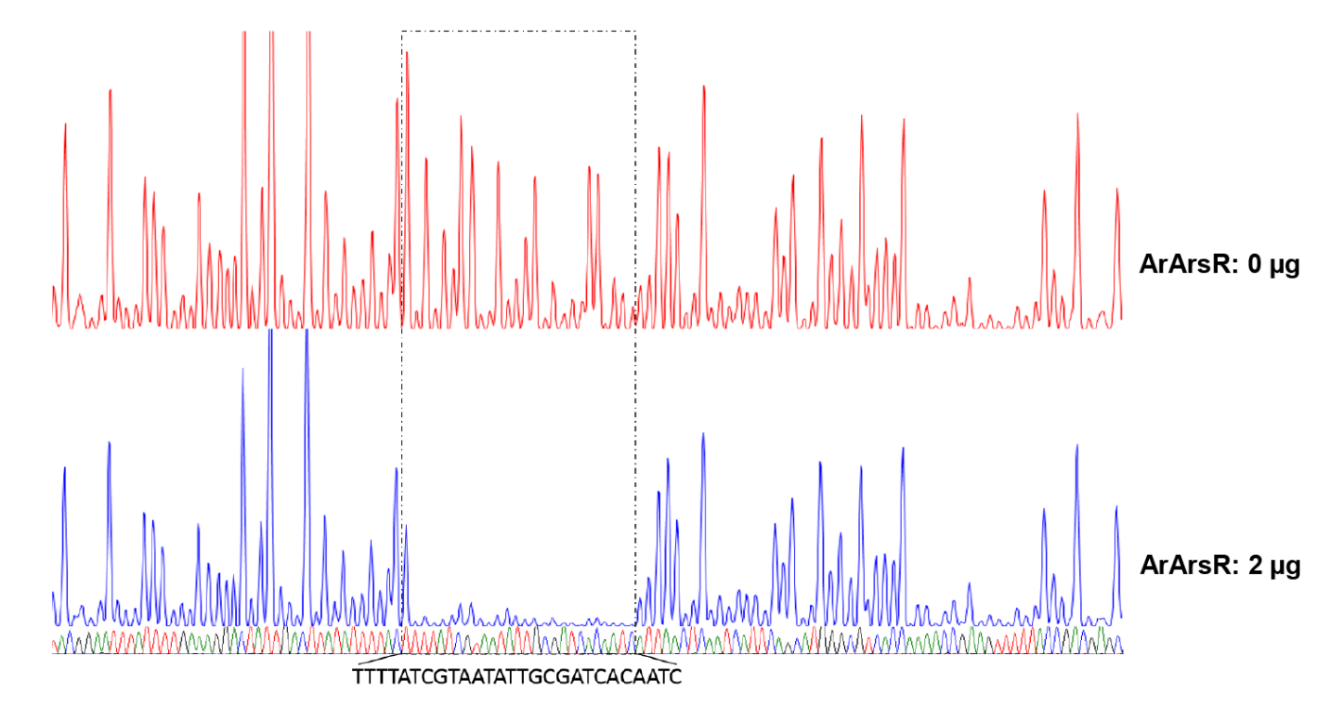


**Fig. S5** Electrophoretic mobility shift assays (EMSA) of ArArsR binding to its own promoter. Lane 1, 1 pmol labelled probes alone; lane 2-7, 1 pmol probes and increasing amount of ArArsR.

**REFERENCES**

1 Yoshinaga M, Cai Y, Rosen BP. Demethylation of methylarsonic acid by a microbial community. *Environ Microbiol*. 2011;13:1205-15.

2 Sambrook J, Russel LD. *Molecular cloning: a laboratory manual*, 3rd edition. Cold Spring Harbor, NY: Cold Spring Harbor Laboratory Press. 2011.

3 Smith GLF, Socransky SS, Smith CM. Rapid method for the purification of DNA from subgingival microorganisms. *Oral Microbiol Immunol*. 1989;4:47-51.

4 Ho SN, Hunt HD, Horton RM, Pullen JK, Pease LR. Site-directed mutagenesis by overlap extension using the polymerase chain reaction. *Gene*. 1989;77:51-9.

5 Zhang J, Wu YF, Tang ST, Chen J, Rosen BP, Zhao FJ. A PadR family transcriptional repressor controls transcription of a trivalent metalloid resistance operon of *Azospirillum halopraeferens* strain Au 4. *Environ Microbiol*. 2022;24:5139-50.

6 Webb B, Sali A. Protein structure modeling with MODELLER. *Methods Mol Biol*. 2021;2199:239-55.

7 Seeliger D, de Groot BL. Ligand docking and binding site analysis with PyMOL and Autodock/Vina. *J Comput Aided Mol Des*. 2010;24:417-22.

8 Prabaharan C, Kandavelu P, Packianathan C, Rosen BP, Thiyagarajan S. Structures of two ArsR As(III)-responsive transcriptional repressors: Implications for the mechanism of derepression. *J Struct Biol*. 2019;207:209-17.

9 DeLano WL. *The PyMOL User’s Manual.* San Carlos, CA: DeLano Scientific. 2001.

10 Peters B, Moad C, Youn E, Buffington K, Heiland R, Mooney S. Identification of similar regions of protein structures using integrated sequence and structure analysis tools. *BMC Struct Biol*. 2006;6:4.

11 Huang K, Chen C, Zhang J, Tang Z, Shen Q, Rosen BP, et al. Efficient arsenic methylation and volatilization mediated by a novel bacterium from an arsenic-contaminated paddy soil. *Environ Sci Technol*. 2016;50:6389-96.

12 Huang K, Xu Y, Zhang J, Chen C, Gao F, Zhao FJ. *Arsenicibacter rosenii* gen. nov., sp. nov., an efficient arsenic methylating and volatilizing bacterium isolated from an arsenic-contaminated paddy soil. *Int J Syst Evol Microbiol*. 2017;67:3186-91.

13 Carlin A, Shi W, Dey S, Rosen BP. The *ars* operon of *Escherichia coli* confers arsenical and antimonial resistance. *J Bacteriol*. 1995;177:981-6.

14 Kovach ME, Elzer PH, Hill DS, Robertson GT, Farris MA, Roop 2nd RM, et al. Four new derivatives of the broad-host-range cloning vector pBBR1MCS, carrying different antibiotic-resistance cassettes. *Gene*. 1995;166:175-6.
